# Agriculture development and CO_2_ emissions nexus in Saudi Arabia

**DOI:** 10.1371/journal.pone.0225865

**Published:** 2019-12-02

**Authors:** Haider Mahmood, Tarek Tawfik Yousef Alkhateeb, Maleeha Mohammed Zaaf Al-Qahtani, Zafrul Allam, Nawaz Ahmad, Maham Furqan

**Affiliations:** 1 College of Business Administration, Prince Sattam bin Abdulaziz University, Alkharj, Saudi Arabia; 2 Kafr Elshiekh University, Kafr Elshiekh, Egypt; 3 College of Education, Prince Sattam bin Abdulaziz University, Al-Dilam, Saudi Arabia; 4 University of Lahore, Lahore, Pakistan; 5 S&P Global Market Intelligence, Islamabad, Pakistan; Xiamen University, CHINA

## Abstract

The agriculture sector may help to improve the environment of any country. The purpose of this research is to test the existence of environmental Kuznets curve (EKC) hypothesis while keeping the energy consumption and agriculture share in income into account and analyze their effects on the CO_2_ emissions per capita of Saudi Arabia. We test both symmetrical, asymmetrical and quadratic effects of agriculture sector on the CO_2_ emissions. An inverted U-shaped relationship between gross domestic product (GDP) per capita and CO_2_ emissions per capita is found. Hence, EKC hypothesis is validated with a turning point at GDP per capita of 77,068 constant Saudi Riyal. Further, a negative and significant effect of agriculture sector on the CO_2_ emissions per capita has been found both in symmetrical and asymmetrical analyses. The magnitudes of effects of increasing and decreasing agriculture share are found statistically different on the CO_2_ emissions, and rising agriculture share in GDP has larger effect than that of decreasing agriculture share. An inverted U-shaped relationship is also found between agriculture share in GDP and CO_2_ emissions per capita with a turning point at 3.22% agriculture share in GDP.

## Introduction

Though economic development in the modern age is substantially dependent on industrialization as well as the use of modern technology. The role of traditional agriculture sector is still significant since it provides a base for the development of an agro-based industry and is a major source of food. Further, the agriculture sector has the potential to assist in protecting the environment from pollution. Industrialization is said to be responsible for displacing the traditional sector as resources are reallocated between segments from the agriculture to the industry [1,2]. Despite these structural changes in many countries, the agriculture sector is still a matter of concern today for many economies as it may have positive or adverse environmental effects. For example, due to production activities, one cannot ignore the negative externalities of agriculture inputs, which may cause environmental degradation through greenhouse gases (GHGs) emissions, e.g. phosphorus and excessive nitrogen [3]. On the other hand, environmental degradation paces up with the transformation of an economy from agriculture to the industrial sector at a macro level while increasing agriculture share in income may protect the environment. Therefore, the agriculture development may encourage a green environment, which ultimately helps to reduce the pollution. Further, agriculture innovation can also play its role to address environmental issues [4]. The technologies and innovation in the agriculture sector can reduce emissions of CO_2_, CH_4_, and N_2_O and can mitigate the sensitivity of GHGs. Cole et al. [5] claimed that due to technological improvement in the agriculture sector, CO_2_ emission is reduced by 32%, biofuel production can offset carbons by 42%, CH_4_ can be reduced by 16% and N_2_O can be reduced by 10%.

At a micro level, government agriculture policies protect the environment. Under the sustainable development programs of Saudi Arabia, various measures have been taken to improve water efficiency, to reduce losses of irrigation water and to apply agronomic measures for sustainable environmental development. Setting a self-sufficiency target caring sound ecologically measures is a big challenge. It should be kept into account that the quality of environment should not be harmed as a consequence of agriculture development. Therefore, agriculture strategy is supposed to be adopted as a measure to promote efficient water usage and ensure a safe and sustainable environment in the Kingdom [6].

A combination of semi-arid to hyper-arid climate exists in Saudi Arabia with very low rainfall rate. The phenomenon of intensive evapotranspiration has led to a significant problem of water scarcity in the country. The percentage of arable land in the country is 2% with significant agriculture crops of fruits and dates. Saudi Arabia, with 2.15 million square kilometers, is demonstrating around 80% of the Arabian Peninsula. It carries 2,410 kilometers of sea coast, 1,760 kilometers goes along with the Red Sea and about 650 kilometers demonstrates eastern coast of the Arabian Sea. Saudi Arabia has a great potential for agriculture, if seawater is cleaned for agriculture purpose. The agriculture strategy in Saudi Arabia promotes the investment with the collaboration of agriculturally potential countries to become self-sufficient in food products which have been imparted in the Vision 2030 [7].

According to the 2017 statistics, 1009 thousands hectors of land has been used for a cultivation purpose in the kingdom and agriculture sector provides jobs to the 550 thousand labor. Cereal, vegetable, fruits and green fodder have been cultivated on 27.6%, 8.1%, 14.4% and 49.9% of total cultivated land area respectively and have been contributed 10.5%, 11.7%, 7.3% and 70.5% of total agriculture production respectively. Moreover, Saudi Arabia is the second-largest exporter of the dates in the world. Considering the importance of agriculture sector, the government of Saudi Arabia is supporting this sector by providing interest-free loans and subsidized inputs. Consequently, the agriculture production of 2017 has been observed twice and eight times larger than the agriculture production of 2000 and 1970 respectively. It shows a rapid increase in agriculture growth but the agriculture share in income has downward trend since 2001 [8].

Saudi Arabia is the most significant country in the Gulf Cooperation Council (GCC) region in the geographical term and also carries most of the population of this region. However, it does not carry an environmentalism history. With industrial growth, environmental issues have been raised. Furthermore, intensive fossil fuel usage creates environmental restraints on the land. To facilitate urbanization and improvisation in living standards, environment quality is sacrificed. Till now, agriculture development in Saudi Arabia has taken significant measure to give pollution-free environment, but industrialization and urbanization added fuel to the fire.

**Fig 1:**
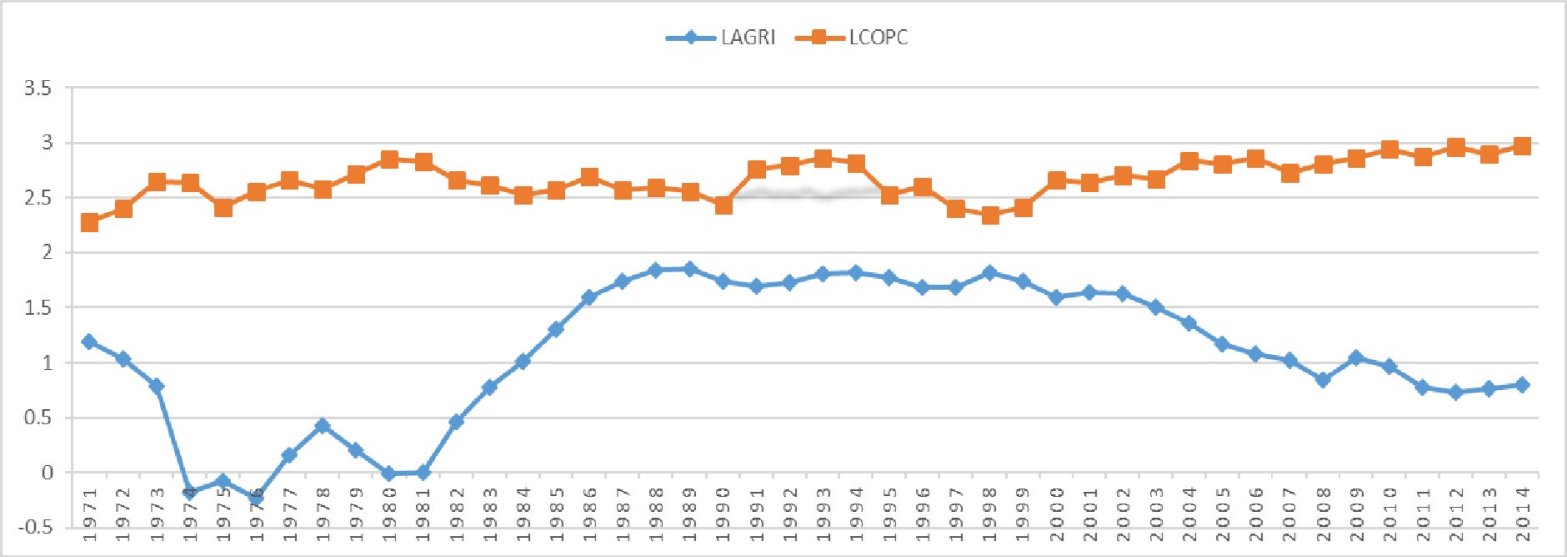
Trends of *LARGI*_*t*_ and *LCOPC*_*t*_.

In the emissions profile of Saudi Arabia, the energy sector majorly contributes CO_2_ emissions as high as about 80% and the rest of the contribution is done by industrial and agriculture sectors. The increasing agriculture sector income share and reducing income share from the industry and/or energy sector can help in controlling pollution emissions in the Kingdom. Fig 1 illustrates the co-movement of the natural logarithm of agriculture share in GDP (*LAGRI*_*t*_) and the natural logarithm of CO_2_ emissions per capita (*LCOPC*_*t*_) during a sample period 1971-2014. Both *LAGRI*_*t*_ and *LCOPC*_*t*_ have a mix of positive and negative trends throughout the period. Further, both series are showing mostly opposite co-movement, it may indicate an expected negative correlation. Therefore, the agriculture sector may assist in protecting the environment by reducing CO_2_ emissions. Further, changes in *LAGRI*_*t*_ are sharper than that of changes in *LCOPC*_*t*_. Moreover, *LAGRI*_*t*_ series shows that declines are comparatively sharper than that of the rises. In this regards, Keynes [9] also argued that declining trends of any macroeconomic variable is sudden and sharper than that of the rising trend. Hence, the asymmetrical effects of macroeconomic variables may be hypothesized. The perception from the co-movement of variables is building that the response of increasing *LCOPC*_*t*_ during decreasing *LAGRI*_*t*_ is comparatively lesser than that of decreasing *LCOPC*_*t*_ during increasing *LAGRI*_*t*_. Therefore, the magnitude of relationship between two in the times of decreasing *LAGRI*_*t*_ is expected lesser than that increasing *LAGRI*_*t*_. Hence, the asymmetrical effects of increasing and decreasing *LAGRI*_*t*_ on the *LCOPC*_*t*_ may be hypothesized from the co-movements of both variables.

Irrespective of sectorial analysis, literature has noticeably considered pollution due to economic development across the globe. Grossman and Krueger [10] established the idea of an inverted U-shaped association between economic development and pollution, and the hypothesis is called the EKC. The literature on how economic development plays a role in the establishment of an environmental profile has extensively been discussed in EKC-related studies. It is assumed that first, development enhances environment degradation. At a later stage, it decreases pollution by embracing environment-friendly technologies. Dinda [11] added this view that in the early stages of development, the industrial sector replaces the agriculture sector that turns out to be the major cause of higher emissions. Later on, clean sectors, e.g. the services sector and/or clean technologies may emerge for rising demand for a clean environment to improve the standard of living at the second stage of development. Therefore, the agriculture sector may play a significant role in tracing the EKC hypothesis. Extensive testing has been done on the existence of EKC hypothesis in recent environment literature [12–15], but testing the role of agriculture sector is relatively scant.

The present study highlights this issue by testing the EKC hypothesis along with the effect of the agriculture sector on CO_2_ emissions to explore whether agriculture development enhances environmental protection or not. Some studies have developed models in the field of plant science, atmospheric science, and agriculture economics to explore the environmental sensitivity of the agriculture sector [3–6,16–17]. However, the macroeconomic effect of the agriculture sector on pollution emissions is relatively scarce in the literature. Moreover, the present study’s objective is to see that agriculture development has symmetric or asymmetric effects on CO_2_ emissions. The testing of asymmetric effects of agriculture sector on the CO_2_ emissions is unexplored in the global environment literature and case of the Saudi Arabian economy as well. This present research claims to have an empirical contribution by testing this issue in Saudi Arabia using the maximum available time range of 1971-2014.

## Literature review

Grossman and Krueger [18] initiated the testing of the EKC hypothesis for forty-two countries with different pollution emissions. They found that pollution is increasing with increasing income at low level of income and is decreasing at higher level of income. Hence, the evidence of EKC hypothesis was proved. Selden and Song [19] investigated the EKC hypothesis for 30 countries with a mixed level of income from 1973-1984. They corroborated the EKC hypothesis by establishing the inverted U-shaped relationship between four types pollution emissions and economic growth. de Bruyn et al. [20] claimed that the existence EKC in the panel testing does not necessarily hold for individual countries’ time series analysis. They investigated the pollution-income relationship for four developed countries UK, USA, Western Germany and Netherlands. They found the positive impact of economic growth and negative impact of structural changes and technological advances on the pollution emissions.

Recent literature also tests the EKC hypothesis in a single-country case and the panel of countries as well. For example, Dogan and Tarkekul [12] scrutinized the existence of an association between carbon dioxide emissions (CDE), financial market development (FMD), trade openness, real income, it's square and urbanization in the USA from 1960-2010. They used the auto-regressive distributive lag (ARDL) and Granger causality approach to check the cointegration and causal relationships. The USA is well-known for vast volume of GHGs emissions, energy consumption and urbanization. The empirical results showed that the prime cause of CO_2_ emission is a higher consumption of energy. Moreover, urbanization is responsible for increasing GHGs emissions in the country. They did not confirm the existence of EKC in the USA. Further, they found bidirectional causality between urbanization and emissions, real income and emissions_,_ trade openness and GDP, urbanization and GDP, energy consumption and emissions. Furthermore, sufficient evidence was found to support unidirectional causality from FMD to output, urbanization to FMD and GDP to energy consumption. In a spatial analysis, Mahmood et al. [14] tested the EKC hypothesis in a group of six East Asian countries. The EKC hypothesis was validated with quadratic effect of income on CDE. Moreover, trade openness, energy intensity and foreign direct investment (FDI) seemed to have positive effects on the CO_2_ emissions. Shujah-Ur-Rahman et al. [15] investigated the EKC hypothesis and the role of FMD in shaping the EKC hypothesis in Pakistan from 1970-2016. They tested the EKC hypothesis with and without interaction variables. They found the negative effect of FMD and corroborated the EKC hypothesis in Pakistan. Further, they reported bi-directional causality between income and CO_2_ emissions and unidirectional causality from FMD to CO_2_ emissions.

Churchill et al. [21] investigated the EKC hypothesis for twenty OECD countries using data from 1870-2014 and caring cross-sectional dependency in the unit root and cointegration tests. They found evidence of EKC in the whole panel. However, the mix pieces of evidence of existence and non-existence of EKC hypothesis are found in the country-specific testing and EKC was found in nine out of twenty countries. Further, the turning points are estimated between 18955$-89540$. Albulescu et al. [22] explored the EKC hypothesis for 14 Latin American countries using data from 1980-2010 and quantile regression analysis. They found a partial evidence of EKC hypothesis in the panel and unclear effect of FDI on pollution.

Some studies also tested the effect of the agriculture sector on pollution emissions. Ullah et al. [16] conducted a study on the relationships between CDE and agricultural ecosystem in Pakistan for a period of 1972-2014. They applied the Johansen and ARDL cointegration tests. The empirical findings showed that CDE and agricultural ecosystem had significant long run relationship. The result showed that a 1% rise in biomass burned crop residue increased the CO_2_ emissions by 1.29%. Similarly, a 1% rise in synthetic fertilizers, cereal production, agricultural machinery, stock of livestock and other crop productions caused to raise the CDE by 0.05%, 0.03%, 0.05%, 0.45% and 0.65% respectively. A bidirectional causality existed among CO_2_ emissions and cereal production, rice area paddy harvested and CO_2_ emissions, and CO_2_ emissions and other crop productions. The results also displayed that agriculture machinery, cereal production, stock of livestock and other crop productions contributed to CDE significantly.

Long et al. [17] explored the factors which influenced the CDE for a period 1997-2014. The results of the study indicated that innovation seemed to discourage CO_2_ emissions, whereas FDI had positive impacts on innovation in China. Moreover, the training of labor and collaboration among academia and agriculture had been essential found to enhance innovation for green agriculture. Ravindra et al. [23] examined the relation between air pollution and burning of agricultural crop residue in India over the financial period of 2003-04 to 2016-17. They observed that the population growth is caused to enhance the demand for food, and it exerted the pressure on crop production; as a result, agricultural crop residue increased. They estimated the numerous atmospheric emissions from crop-residue burning and found that GHGs (CO_2_, CH_4_, N_2_O) from burning were equal to the 211 Tg/yr.

Leitao [24] examined the relationship between the productivity of agriculture and CDE. With the help of a time series data analysis from 1960 to 2015 in Portugal, Granger causality has been used to test some theoretic hypotheses which clarified the bidirectional causality among climate change and the productivity of agriculture. The results showed that land productivity, agricultural labor and agricultural raw material exports impacted CDE in the country positively. Hence, these factors could increase the intensity of environmental pollution. Chandio et al. [25] analyzed how agriculture and financial development can impact CO_2_ emission by incorporating the variables of energy consumption from the agriculture sector and FDI in Pakistan during 1980-2016. They applied the ARDL cointegration technique along with other cointegration tests and found that a rise in FDI and financial development caused to enhance the environmental quality. Furthermore, environmental quality could be improved by using more electricity in the agriculture sector and higher income levels as well. Ahmad et al. [26] examined the effect of industrial growth and population growth on CO_2_ emissions of Bangladesh, India, Nepal and Pakistan. They found that both industrial and population growth positively affected the CO_2_ emissions in each country estimation and as a panel as well. Therefore, industrialization could be counted for environmental degradation in the mentioned countries.

Environmental literature also signified the importance of asymmetric analysis to a full extent. At first, Shahbaz et al. [27] initiated the asymmetrical analyses of FMD and income on the environmental quality in Pakistan and found asymmetries in most of the effects. They used quarterly data for the analysis of the period from 1985-2014. They used an extensive index of FMD developed by using different indicators. The findings of this study indicated that using energy in an unproductive way could lead to higher emissions which raised the need to introduce more advanced technology. It could also have helped to conserve energy while also assisted in making production more efficient. With the help of this efficient technology, not only the consumption of energy could be made more sustainable, but the quality of the environment could also be improved for a longer-term by boosting up more advanced production procedures. Moreover, the bank-based financial development was seen to be deterring the environment as well. They recommended that the government ought to allot financial resources for environment-friendly businesses and hearten the lenders by providing them funds for the energy sector instead of dissipating them in consumer financing. Using a period 1971-2014, Mahmood et al. [13] investigated the asymmetric effect of trade openness on the CDE and tested the EKC hypothesis in Tunisia. They found a valid EKC in Tunisia and also found that increasing trade openness had environmentally degrading effect while decreasing trade openness had an insignificant effect. Alkhateeb and Mahmood [28] investigated the asymmetric relationships among energy consumption, trade openness and economic growth in Egypt. They found that no matter if economic growth improved or declined, energy consumption was seen to increase. Additionally, the magnitude of effects of the energy consumption on the environment was asymmetric. Further, only increasing trade openness was found to contribute to higher energy consumption while impact of decreasing trade openness was insignificant.

In the pollution literature of Saudi Arabia, Mahmood and Alkhateeb [29] explored how the income level and trade have an impact on CDE by covering period 1970-2016. They applied the unit root test and cointegration. The unit root test showed the integration level as one, whereas the cointegration had been found through the bound test. The results showed that higher income increases CDE while its square term hurts CDE in the long-term. Consequently, the EKC hypothesis had been found. Furthermore, the effect of trade was found negative on the CDE, and for that reason, pollution levels had been decreasing through the help of trade. They suggested that the government of Saudi Arabia should liberalize the trade to safeguard the environment.

Mahmood et al. [30] investigated the EKC hypothesis in Saudi Arabia from 1971-2014 and tested the asymmetric effects of FMD and energy consumption on CO_2_ emissions. The existence of EKC was proven, and negative asymmetric effect of FMD on the CO_2_ emissions was also seen in the findings. Moreover, decreasing FMD and energy consumption was found helpful in reducing CDE. However, the effect of increasing FMD and energy consumption were found insignificant. Using a period 1980-2017, Alsamara et al. [31] explored and corroborated the EKC hypothesis in the GCC panel and also in the country analysis of all GCC countries except Oman. Further, FMD had negative and energy consumption and exports had the positive effect on CO_2_ emissions. They also found Granger causality from income per capita to pollution emissions. Using period 1971-2014, Raggad [32] investigated the EKC hypothesis, and the results indicated that income has a monotonic and positive effect on the CDE. Energy use and urbanization were seen to have a positive and negative impact on CO_2_ emissions respectively. Table 1 shows the summary of all reviewed literature.

**Table 1:** Literature summary.

Authors	Data Coverage	Region	Methods	Major Findings
Grossman and Krueger [18]	1977-1988	42 countries	Fixed effects (FE) and random effects (RE)	Pollution is increasing with increasing income at a low level of income and vice versa. EKC hypothesis was proved.
Selden and Song [19]	1973-1984	30 countries	FE and RE	The EKC hypothesis was found in relationships of 4 types of pollution emissions and economic growth.
de Bruyn et al. [20]	1960-1993	UK, USA, Western Germany and the Netherlands	Reduced form Regression	Pollution emissions are positively related to economic growth and negatively related to structural changes and technological advances.
Dogan and Tarkekul [12]	1960-2010	USA	ARDL and Granger causality	EKC was not found. The CO_2_ emission is caused by energy consumption, urbanization, real income, trade openness and energy consumption.
Mahmood et al. [14]	1991-2014	6 East Asian countries	Spatial FE and RE	EKC was validated. Spillover and local effects of FMD, FDI, trade openness were found on CO_2_ emissions.
Shujah-Ur-Rahman et al. [15]	1970-2016	Pakistan	ARDL and Granger causality	EKC was found. FMD has negative effect on CO_2_ emissions. Bi-directional causality was found between income and CO_2_ emissions.
Churchill et al. [21]	1870-2014	20 OECD countries	Panel cointegration tests	EKC holds in the panel and 9 out of 20 countries’ time series analyses.
Albulescu et al. [22]	1980-2010	14 Latin American countries	Quantile regression analysis	Partial evidence of EKC hypothesis was found and unclear effect of FDI on the pollution was found.
Ullah et al. [16]	1972-2014	Pakistan	Cointegration tests	Biomass burned crop had positive effect on pollution emissions. Bidirectional causality between CO_2_ emissions and most of crops analyzed.
Long et al. [17]	1997-2014	China	First and second-stage least square	FDI positively affected innovation and innovation reduced the CO_2_ emissions.
Ravindra et al. [23]	2003-04 to 2016-17	India	Ratio and identities	Burning of agricultural crop residue positively contributed to GHGs emissions.
Leitao [24]	1960-2015	Portugal	Granger causality	Labor, land productivity and raw material exports positively contributed to CO_2_ emissions and bidirectional causality among climate change and the productivity of agriculture was found.
Chandio et al. [25]	1980-2016	Pakistan	ARDL	FDI and financial development negatively affected the CO_2_ emissions in the agriculture sector.
Ahmad et al. [26]	1980-2008	Bangladesh, India, Nepal and Pakistan	Cointegration	Industrial and population growth positively affected the CO_2_ emissions.
Shahbaz et al. [27]	1985Q1- 2014Q4	Pakistan	Nonlinear ARDL	Energy consumption and FMD had the positive and asymmetrical effects on the CO_2_ emissions.
Mahmood et al. [13]	1971-2014	Tunisia	Nonlinear ARDL	EKC was found valid. Increasing and decreasing trade openness has the positive and insignificant effects on CO_2_ emissions respectively.
Alkhateeb and Mahmood [28]	1971-2014	Egypt	Nonlinear ARDL	Economic growth and increasing trade openness had positive effects on the energy consumption.
Mahmood and Alkhateeb [29]	1970-2016	Saudi Arabia	ARDL	EKC was found valid and trade openness had a negative effect on the CO_2_ emissions
Mahmood et al. [30]	1971-2014	Saudi Arabia	Nonlinear ARDL	EKC was found valid and decreasing FMD and energy consumption helped in decreasing CO_2_ emissions.
Alsamara et al. [31]	1980-2017	GCC region	Panel cointegration and causality	EKC was found valid and FMD had negative and energy consumption and exports had positive effects on CO_2_ emissions.
Raggad [32]	1971-2014	Saudi Arabia	ARDL	EKC was not found and energy use (urbanization) show a positive (negative) effects on CO_2_ emissions.

The reviewed literature signifies the importance of the agriculture sector in the environment. Further, the effect of agriculture is not certain, and it is an empirical question for any country and testing environmental effects of the agriculture sector is missing in the Saudi literature. Further, previous Saudi literature showed the mix evidence of the EKC hypothesis. The present study is exploring, for the first time, the role of the agriculture sector in shaping the EKC in Saudi Arabia. Moreover, it considers the possible asymmetrical environmental effects of agriculture sector for a claim of contribution in Saudi literature.

## Methodology

The EKC hypothesis can be tested by regressing the pollution emissions on income and income square. The positive and negative impacts of income and income square on the pollution emissions respectively may corroborate the EKC hypothesis. The effect of income is seen to be quadratic on CO_2_ emission pollution. The scale effect represents the positive association between income and pollution emissions. Because, energy demand is increasing with the increasing income level at the first stage of growth which accelerates the pollution emissions. In the first phase, energy consumption has a profound role to play. Later on, a clean environment is demanded with further increasing economic growth. Therefore, a negative association is expected between income and pollution emissions in the second phase of EKC. Dinda [11] elaborates that environmental degradation starts once a country moves from agriculture to the industrial sector. It means that with more agriculture sector share, one may expect lower levels of pollutant emissions. Therefore, the basic structure of the economy matters a lot for its environmental performance. Particularly, the agriculture sector may play a significant role in protecting the environment. To test the effects of income, energy consumption and agriculture sector on the pollution emissions, we hypothesize the following model:

$$LCOPC_{t}=f(LGDPC_{t},LGDPC_{t}^{2},LAGRI_{t},LECPC_{t})$$ (1)

Where,

*LCOPC*_*t*_ = natural logarithm of CO_2_ emissions per capita

*LGDPC*_*t*_ = natural logarithm of GDP per capita in constant Saudi Riyal

*LGDPC*_*t*_^*2*^ = square of *LGDPC*_*t*_

*LAGRI*_*t*_ = natural logarithm of percentage share of agriculture value added in the GDP

*LECPC*_*t*_ = natural logarithm of energy consumption per capita

*t* = annual period of 1971-2014

All variables are obtained from the World Bank [33] and are converted into natural logarithm form to estimate elasticity. The data are provided in supplementary information as S1 File. Data series. (XLSX). *LGDPC*_*t*_ and *LGDPC*_*t*_^*2*^ are likely to have positive and negative coefficients respectively to proof the EKC hypothesis. The positive and negative effects of agriculture share and energy consumption per capita on the CO_2_ emissions are expected respectively. To estimate the hypothesized model, we need to examine the integration level in the model. We are using the Ng and Perron [34] unit root test which is suitable in the small sample case. Further, we are using linear ARDL of Pesaran et al. [35] to estimate the effects of hypothesized variables on the CO_2_ emissions. ARDL cointegration is chosen as it provides efficient estimates even in a mixed integration order due to the bound testing procedure. The bound testing procedure depends on lower bound assuming level stationary variables I(0) and on upper bound assuming first difference stationary I(1) variables. Therefore, it is efficient in case of a mixed order of integration. It solves the potential problem of endogeneity in the model through auto regressive process and provides consistent estimates. Further, it has capacity to choose different optimum lag length for each variable so it saves the degree of freedom in parsimonious way as compare to other cointegration methods [35]. The linear ARDL form of our model is as follows:

$$\begin{array}{l} \Delta LCOPC_{t}=\alpha_{0}+\alpha_{1}LCOPC_{t-1}+\alpha_{2}LGDPC_{t-1}+\alpha_{3}LGDPC_{t-1}^{2}+\alpha_{4}LAGRI_{t-1}^{}\\ +\alpha_{5}LECPC_{t-1}^{}+\sum_{j=1}^{m1}\delta_{1j}\Delta LCOPC_{t-j}+\sum_{j=0}^{m2}\delta_{2j}\Delta LGDPC_{t-j}+\sum_{j=0}^{m3}\delta_{3j}\Delta LGDPC_{t-i}^{2}\\ +\sum_{j=0}^{m4}\delta_{4j}\Delta LAGRI_{t-j}+\sum_{j=0}^{m5}\delta_{5j}\Delta ECPC_{t-j}+\psi_{t} \end{array}$$ (2)

Eq 2 can be tested for the existence of cointegration after choosing the optimum lag lengths (m_i_) for each variable. Afterwards, bound test is applied on the H_0_ of no-cointegration, *a*_*1*_*= a*_*2*_*= a*_*3*_*= a*_*4*_*= a*_*5*_*=0*. A long run relation in the model can be claimed if the estimated F-value from the bound test is larger than upper critical value. We utilized Kripfganz and Schneider’s [36] critical values which are even efficient in case of a small sample. Hereafter, we calculate the long run effects through normalized coefficients of lagged-level variables. Replacing the error correction term (*ECT*_*t-1*_) with a lagged-leveled variable in Eq 2, the short run effects would be discussed from the coefficients of lagged-differenced variables. After estimating the symmetrical effect of agriculture share on the CO_2_ emissions, we test the asymmetry of agriculture share. Keynes [9] claimed the decreasing trend suddenly shifts from the increasing trend of the macroeconomic series sharply. Therefore, the increasing component and decreasing component of variable don’t necessarily have the same effects. Considering this argument, we assume the asymmetrical effects of agriculture share on the CO_2_ emissions. Following Shin et al. [37], we split the *LAGRI*_*t*_ into two series in following way:

$$LAGRIP_{t}^{}=\sum_{i=1}^{t}\Delta LAGRI_{i}^{+}=\sum_{i=1}^{t}\max(\Delta LAGRI_{i}^{},0)$$ (3)

$$LAGRIN_{t}^{}=\sum_{i=1}^{t}\Delta LAGRI_{i}^{-}=\sum_{i=1}^{t}\min(\Delta LAGRI_{i}^{},0)$$ (4)

*LAGRIP*_*t*_ and *LAGRIN*_*t*_ are the partial sum of positive and negative deviations in *LAGRI*_*t*_ and showing positive and negative variables of agriculture share respectively. Replacing *LAGRI*_*t*_ with *LAGRIP*_*t*_ and *LAGRIN*_*t*_ in Eq 2, non-linear ARDL is expressed as follows:

$$\begin{array}{l} \Delta LCOPC_{t}=\beta_{0}+\beta_{1}LCOPC_{t-1}+\beta_{2}LGDPC_{t-1}+\beta_{3}LGDPC_{t-1}^{2}+\beta_{4}LAGRIP_{t-1}^{}\\ +\beta_{5}LAGRIN_{t-1}^{}++\beta_{6}LECPC_{t-1}^{}+\sum_{j=1}^{n1}\gamma_{1j}\Delta LCOPC_{t-j}+\sum_{j=0}^{n2}\gamma_{2j}\Delta LGDPC_{t-j}\\ +\sum_{j=0}^{n3}\gamma_{3j}\Delta LGDPC_{t-j}^{2}+\sum_{j=0}^{n4}\gamma_{4j}\Delta LAGRIP_{t-j}+\sum_{j=0}^{n5}\gamma_{5j}\Delta LAGRIN_{t-j}+\\ \sum_{j=0}^{n6}\gamma_{6j}\Delta ECPC_{t-j}+\psi_{t} \end{array}$$ (5)

Eq 5 is used to estimate the nonlinear ARDL results using the same procedure proposed by Pesaran et al. [35] in linear ARDL, discussed before for Eq 2. Afterwards, the Wald test is employed to test the asymmetrical effects of agriculture share.

## Results and discussions

The precondition for any time series analysis is that series should not have unit root problem. Table 2 shows the results of Ng and Perron [34] unit root test. It can be observed from the Table 2 that all the variables of our hypothesized model are non-stationary at their levels. At their first differences, all test statistics corroborate the stationarity of all variables at the 5% level of significance except *LECPC*_*t*_ which is stationary at 10%. Therefore, the model is fine to proceed for cointegration analysis.

**Table 2:** Unit root test’s results.

Variables	MZa	MZt	MSB	MPT
*LCOPC*_*t*_	-13.7765	-2.6137	0.1897	6.6769
*LGDPC*_*t*_	-3.1465	-1.2507	0.3975	28.8770
*LAGRI*_*t*_	-6.0584	-1.7171	0.2834	15.0183
*LAGRIP*_*t*_	-4.1588	-1.3332	0.3206	20.7900
*LAGRIN*_*t*_	-4.1447	-1.4281	0.3421	21.6524
*LECPC*_*t*_	-2.7809	-1.1281	0.4057	31.1944
*ΔLCOPC*_*t*_	-20.7058**	-3.2054**	0.1548**	4.4746**
*ΔLGDPC*_*t*_	-20.3545**	-3.1889**	0.1567**	4.4848**
*ΔLAGRI*_*t*_	-20.1978**	-3.1768**	0.1573**	4.5181**
*ΔLAGRIP*_*t*_	-18.1948**	-3.0158**	0.1658**	5.0105**
*ΔLAGRIN*_*t*_	-18.5324**	-3.0450**	0.1638**	4.9255**
*ΔLECPC*_*t*_	-15.3313*	-2.7683*	0.1806*	5.9463*

Note:

** and * show stationarity at 5% and 10% level of significance. MPT, MSB, MZt and MZa are modified versions of PT, SB, Zt and Za test respectively.

Table 3 shows the results of model 1 of linear ARDL discussed in Eq 2 and of model 3 of non-linear ARDL discussed in Eq 5. We also regress the model 2 by assuming the quadratic effect of agriculture sector on the CO_2_ emissions. At first, we apply the bound test on the models to confirm the existence of cointegration. The calculated F-values are found larger than upper bound critical values at 10% and 5% in model 1 and model 3, respectively. So, the cointegration is proven in models 1 and 3 but F-value of model 2 do not corroborate the cointegration in the model. Another way to test cointegration and short-run relationship is to observe the coefficient of *ECT*_*t-1*_ [35] and estimated coefficients of *ECT*_*t-1*_ are found negative and statistically significant in all three models. Therefore, we corroborate the long run and short-run relationships in all models. Further, the robustness tests of heteroscedasticity, serial correlation, functional form and normality are applied. The estimated *F-Hetro*, *F-Serial*, *F-RESET* and *χ*^*2*^*-Normality* are found reasonably small, and p-values are found more than 0.1 in all models. So, our estimated models are out of any econometric problem and are suitable for interpretations.

**Table 3:** Estimates from linear and nonlinear ARDL models.

Variables	Model 1	Model 2	Model 3
Long Run Results			
*LGDPC*_*t*_	31.0937* (0.0725)	0.6835** (0.0160)	34.2187* (0.0890)
*LGDPC*_*t*_^*2*^	-1.3787* (0.0719)		-1.5205* (0.0878)
*LAGRI*_*t*_	-0.2424** (0.0249)	0.3404* (0.0802)	
*LAGRI*_*t*_^*2*^		-0.1457* (0.0931)	
*LAGRIP*_*t*_			-0.3153* (0.0922)
*LAGRIN*_*t*_			-0.1998* (0.0857)
Wald Test			3.6749* (0.0651)
*LECPC*_*t*_	0.1282 (0.3101)	0.3256*** (0.0000)	0.3376 (0.1464)
Intercept	-173.3340* (0.0740)	-7.8576** (0.0316)	-192.4240* (0.0888)
Short Run Results			
*ΔLCOPC*_*t-1*_	0.1736 (0.1425)		
*ΔLGDPC*_*t*_	1.0786 (0.8699)	0.3916** (0.0142)	0.0659 (0.9938)
*ΔLGDPC*_*t*_^*2*^	-0.0189 (0.9482)		0.0317 (0.9324)
*ΔLGDPC*_*t-1*_^*2*^			0.0109 (0.1945)
*ΔLAGRI*_*t*_	-0.0031 (0.9689)	0.1950** (0.0263)	
*ΔLAGRI*_*t*_^*2*^		-0.0835** (0.0295)	
*ΔLAGRIP*_*t*_			0.2084 (0.2514)
*ΔLAGRIN*_*t*_			-0.0997* (0.0955)
Wald Test			3.3403* (0.0772)
*ΔLECPC*_*t*_	0.7247*** (0.0000)	0.1865*** (0.0028)	0.7769*** (0.0003)
*ECT*_*t-1*_	-0.5672*** (0.0000)	-0.5729*** (0.0000)	-0.4988*** (0.0005)
Bound Test Estimated F-Value	3.1783*	2.6883	3.4112**
Critical Bound F-values with level of significance			
At 1%	3.2778-4.3109	3.2778-4.3109	At 1% 3.0379-4.1121
At 5%	2.5448-3.4712	2.5448-3.4712	At 5% 2.3851-3.3551
At 10%	2.2001-3.0679	2.2001-3.0679	At 10% 2.0766-2.9892
Diagnostic tests			
*F-Hetro*	1.6498 (0.1371)	1.1814 (0.3364)	1.4374 (0.2060)
*F-Serial*	0.0316 (0.9690)	1.7864 (0.1821)	0.1768 (0.8388)
*F-RESET*	0.4971 (0.4860)	0.0232 (0.8798)	0.0003 (0.9856)
*χ*^*2*^*-Normality*	0.7292 (0.6945)	2.2954 (0.3173)	3.3710 (0.1854)

Note: () carries probability values.

*, ** and *** show the statistical significance at 10%, 5% and 1% respectively.

In addition to the diagonostic tests in Table 3, we also test the unit problem in the residuals’ series by applying the ADF test with a specification of no intercept and no trend. The results of ADF test in Table 4 show that residuals from all models 1-3 are stationary at level at 1% level significance. Hence, there is no issue of unit root problem in the residuals of estimated models.

**Table 4:** Residuals from estimated models.

Model	ADF test on level of residuals [without intercept and trend]
1	-7.0880 ***
2	-5.5864***
3	-6.3007***

Note:

*** shows stationarity at 1% level of significance.

In the long run results, models 1 & 3 confirm the existence of the EKC due to positive and negative coefficients of *LGDPC*_*t*_ and *LGDPC*_*t*_^*2*,^ respectively. Further, the turning points of inverted U-shaped curve are found at 78941 (exponent of 31.0937/2/1.3787) and 77068 (exponent of 34.2187/2/1.5205) of GDP per capita constant Saudi Riyals in model 1 and model 3 respectively. Moreover, we found an inelastic positive effect of GDP per capita on CO_2_ emissions per capita in model 2.

In the relationship of agriculture share (*LAGRI*_*t*_) and CO_2_ emissions per capita, the coefficient of *LAGRI*_*t*_ shows a negative effect with elasticity less than one in model 1. A negative effect shows that increasing agriculture share in the GDP has positive environmental effects as increasing agriculture share may help in reducing CO_2_ emissions per capita. The elasticity coefficient explains that a 1% increase in agriculture share percentage of GDP helps in reducing 0.2424% of CO_2_ emissions per capita. In model 2, the quadratic effect of *LAGRI*_*t*_ is also tested to verify the U-shaped, inverted U-shaped or monotonic relationship between *LAGRI*_*t*_ and *LCOPC*_*t*_. We found the positive and negative effects of *LAGRI*_*t*_ and *LAGRI*_*t*_^*2*^ on the CO_2_ emissions respectively. Hence, the inverted U-shaped relationship is proved between agriculture sector and CO_2_ emissions with a turning point at 3.22% agriculture share to GDP (exponent of 0.3404/2/0.1457). The average agriculture share to GDP is calculated and found at 3.56% in the sample period 1971-2014 which is more than 3.22%. Hence, this relationship is found at second stage of inverted U-shaped curve after a point of inflection (3.22%) and we may conclude that agriculture sector has negative effect on the CO_2_ emissions in the sample period.

In the asymmetry analysis, the coefficients of *LAGRIP*_*t*_ and *LAGRIN*_*t*_ are found negative and significant in model 3. But, the magnitudes of the coefficients of *LAGRIP*_*t*_ and *LAGRIN*_*t*_ seems different in long run. To verify, the Wald test on the H_0_ of symmetrical effects of *LAGRIP*_*t*_ and *LAGRIN*_*t*_ is applied. It is rejected at 10% level and validates the asymmetrical impacts of *LAGRIP*_*t*_ and *LAGRIN*_*t*_ on the CO_2_ emissions per capita. The elasticity parameter of *LAGRIP*_*t*_ is found larger than that of elasticity parameter of *LAGRIN*_*t*_. It means that increasing agriculture share in GDP can have larger negative effect on the CO_2_ emissions per capita than the decreasing agriculture share. Moreover, 1% increase and 1% decrease in agriculture share are responsible for 0.3153% decreasing and 0.1998% increasing CO_2_ emissions per capita, respectively. After confirming the asymmetry, we conclude the superiority of nonlinear ARDL model’ results because ignoring a statistically significant asymmetry in the model may count for a model specification biasness. Hence, we conclude the turning point of the EKC at GDP per capita of 77068 constant Saudi Riyal. Lastly, energy consumption has statistically insignificant in models 1 and 3 and has positive and statistically significant effect in model 2.

In the short-run estimates, the effects of *LGDPC*_*t*_ and its square are found insignificant in model 1 & 3 but a positive effect *LGDPC*_*t*_ is found statistically significant in model 2. The symmetrical effect of agriculture share (*LAGRI*_*t*_) and asymmetrical effect of increasing agriculture share (*LAGRIP*_*t*_) are found insignificant in model 1 and model 3, respectively. Nevertheless, the asymmetrical negative impact of decreasing agriculture share (*LAGRIN*_*t*_) is found significant, and a 1% decreasing agriculture share is found responsible for increasing 0.0997% of CO_2_ emissions. In addition, the Wald test corroborates asymmetry by rejecting the null hypothesis of symmetry in the short run. Inverted U-shaped relationship is found between agriculture share and CO_2_ emissions in model 2. In last, the impact of energy consumption per capita (*LECPC*_*t*_) is positive in all estimated models.

## Conclusions

The agriculture sector can play a helpful role in improving the environment by reducing GHGs emissions. In this study, the effects of agriculture sector and income are analyzed on CO_2_ emissions, and the purpose is to validate the EKC hypothesis including the role of the agriculture sector in establishing the environmental profile of the Kingdom. For this purpose, we test the symmetrical, quadratic and asymmetrical impacts of agriculture share on the CO_2_ emissions per capita, after testing the order of integration. The bound testing procedure corroborates the cointegration and asymmetrical effect of agriculture share was found valid in Wald test in the long and short run. Therefore, an estimated model with asymmetrical effect of agriculture share is most reliable to conclude the results.

The EKC hypothesis is validated in the relationship of GDP per capita and CO_2_ emissions with a turning point at 77068 of GDP per capita constant Saudi Riyal. Further, we found the negative asymmetrical impact of agriculture share on the CO_2_ emissions per capita. The asymmetry is corroborated in the magnitude of effects of increasing and decreasing agriculture share on the CO_2_ emissions per capita. The effect of increasing agriculture share is found larger than the decreasing share. In whole, we conclude that increasing agriculture sector share in the economy has positive environmental effects by reducing CO_2_ emissions per capita and decreasing agriculture sector share is responsible for environmental degradation. In the long run, energy consumption does not have any impact but in a short-run, this effect is seen to be significant.

The asymmetrical effect of increasing agriculture share is found insignificant in the short run. However, decreasing agriculture share significantly increases the emissions and asymmetry is also proved in the short run as well. All in all, a negative relationship is shown between the agriculture sector and CO_2_ emissions which indicated that higher agriculture sector activities can help to reduce emissions and to improve the environment in the country and vice versa.

## Supporting information

S1 File Data series. (XLSX)
